# A short history of pluripotent stem cells markers

**DOI:** 10.1016/j.stemcr.2023.11.012

**Published:** 2023-12-28

**Authors:** Peter W. Andrews, Paul J. Gokhale

**Affiliations:** 1 The School of Biosciences, The University of Sheffield, Western Bank, Sheffield S10 2TN, UK

## Abstract

The expression of one or more of a small number of molecules, typically cell surface-associated antigens, or transcription factors, is widely used for identifying pluripotent stem cells (PSCs) or for monitoring their differentiation. However, none of these marker molecules are uniquely expressed by PSCs and all are expressed by stem cells that have lost the ability to differentiate. Consequently, none are indicators of pluripotency, per se. Here we summarize the nature and characteristics of several markers that are in wide use, including the cell surface antigens, stage-specific embryonic antigen (SSEA)-1, SSEA-3, SSEA-4, TRA-1-60, TRA-1-81, GCTM2, and the transcription factors POUF5/OCT4, NANOG, and SOX2, highlighting issues that must be considered when interpreting data about their expression on putative PSCs.

## Teaser

This review summarizes the nature and expression patterns of several markers widely used for identifying and monitoring PSCs, highlighting issues that must be considered when interpreting data about their expression by putative PSCs. Specifically, it points out that no markers are exclusively expressed by PSCs and none can be used to definitely prove pluripotency.

## Introduction

While cell differentiation involves extensive changes in patterns of gene expression, it is commonly convenient to monitor developing systems by following the expression of just a small number of gene products that act as markers, or indicators, of particular cell types or stages of differentiation. Although many molecules that are differentially expressed by pluripotent stem cells (PSCs) and somatic cells can be used as markers in particular contexts, there are a few that have become well established and are in wide use for identifying PSCs and for monitoring their differentiation (International Stem Cell Initiative, 2007). Some, often complex carbohydrate structures, are expressed on the cell surface and are of particular utility since they are readily detected on living single cells. They can be used not only to monitor, but also to manipulate populations of cells for functional studies. Others are intracellular proteins, including several transcription factors that play key roles in controlling the phenotype of the PSCs and their ability to differentiate in response to specific cues.

However, it is doubtful if any single gene product is uniquely expressed by only one cell type. Certainly, none of the markers commonly used for PSCs are only expressed by cells that exhibit pluripotency. Each offers advantages and disadvantages in different contexts but a failure to appreciate the nature of a particular marker and its wider pattern of expression can lead to inappropriate or questionable conclusions, an issue raised in the recent report of the ISSCR on “Standards for Human Stem Cell Use in Research” (https://www.isscr.org/standards). In this review, we consider the history of a number of the commonly used PSC markers highlighted in that review, summarizing their characteristics and expression patterns and highlighting not only their utility, but also potential limitations and pitfalls when used to identify and monitor these cells.

## Cell surface antigens

Studying the immune system in the 1960s, Ted Boyse and Lloyd Old coined the term differentiation antigen to describe cell surface antigens marking different cells at successive stage of development of the hematopoietic system (Boyse and Old, 1969). An important facet of such antigens is that they not only mark particular cells, but also provide tools for their isolation allowing analysis of their function and position in a developmental hierarchy. At that time, before the advent of monoclonal antibodies, Boyse and Old used genetic analysis of allelic variants of the antigens they defined to overcome the inherent problems of antisera that contain multiple antibodies of diverse specificities, which may vary from batch to batch. Among the differentiation antigens of lymphocytes they described were Thy1 and Ly-1, -2, and -3, which were used to delineate the development of T lymphocytes, with killer and helper functions (Kisielow et al., 1975).

In parallel with studies of the hematopoietic system, developmental biologists in the 1960s and 1970s sought to exploit the discovery by Leroy Stevens that testicular teratomas are regularly produced in strain 129 mice (Stevens, 1967) to establish models of early embryonic development. Teratomas are germ cell tumors that contain differentiated somatic cells corresponding with all three germ layers, endoderm, mesoderm and ectoderm (Damjanov and Andrews, 2016). They may also contain embryonal carcinoma (EC) cells that are capable of recapitulating cell differentiation occurring in the early embryo, albeit in a disorganized way, to produce derivative cells of all three germ layers (Kleinsmith and Pierce, 1964). Teratomas that also contain EC cells are highly malignant and have been historically distinguished as teratocarcinomas (Damjanov and Andrews, 2007). Studies of EC cells (Solter, 2006) paved the way for the subsequent culture of both murine and human embryonic stem (ES) cells isolated directly from the early embryo (Evans and Kaufman, 1981; Martin, 1981; Thomson et al., 1998) and, eventually, induced pluripotent stem (iPS) cells by reprogramming of somatic cells (Takahashi and Yamanaka, 2006; Takahashi et al., 2007; Yu et al., 2007). It is notable, though, since it has relevance for our later discussion, that some EC cells in tumors and in culture seem to have lost the capacity for differentiation, perhaps because this provides for a more aggressive malignant phenotype. These were designated as nullipotent stem cells (Bernstine et al., 1973; Martin and Evans, 1975; Andrews et al., 1980). To date, no nullipotent ES or iPS cells have been reported, although some do show marked biases in their differentiation capacity (International Stem Cell Initiative, 2018; Koyanagi-Aoi et al., 2013).

Building on the work of Boyse and Old with the hematopoietic system, and reasoning that adult mice should not be tolerant to antigens expressed exclusively in the early embryo, Karen Artzt, working with François Jacob, found that adult strain 129 mice immunized with EC cells of the syngeneic F9 cell line produced an antiserum that identified an antigen expressed in common by murine EC cells and embryonic cells of the mouse blastocyst, but absent from differentiated cells in the later embryo (Artzt et al., 1973). Initial ideas were that this F9 antigen might play a significant role in controlling early embryonic development (Artzt et al., 1974). Unfortunately, these anti-EC cell sera lacked the genetic controls offered by the allelic variants of the differentiation antigens of the hematopoietic system, so that analysis of the embryonic antigens detected was fraught with difficulties (Gachelin et al., 1982). Nevertheless, this early work laid the conceptual framework for the later definition and characterization of a series of embryonic cell surface antigens that were much better defined using monoclonal antibody technology.

## Blood group-related antigens, stage-specific embryonic antigen-1, -3, -4, and -5

**Figure 1:**
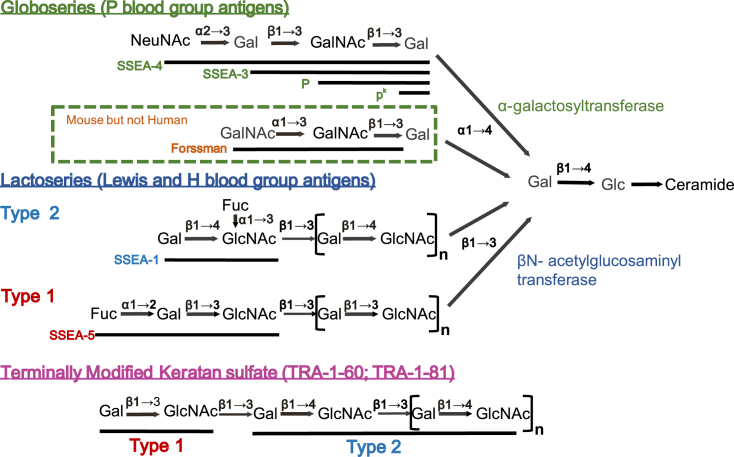
Carbohydrate cell surface antigens of PSCs The cell surface of human and mouse PSC is characterized by the expression of extended polysaccharides of the globo- and lactoseries. These are typically present as glycosphingolipids initiated by sequential monosaccharide addition to a core, lactosyl ceramide: the first addition of galactose initiates the globoseries structures, whereas the first addition of N-acetylglucosamine initiates the formation of poly N-acetyl-lactosoamine chains of the lactoseries structures. The poly N-acetyl-lactosamine chains may be type 1, if the repeating N-acetyl-lactosamine disaccharides have a Galβ1→3GlcNAc linkage, and type 2 if a Galβ1→4GlcNAc linkage. As well as being associated with glycolipids, the lactoseries structures may also be linked to glycoproteins. Among these, the extended type 2 poly-N-acetyl-lactosamine may be sulfated to form keratan sulfate. The epitopes of the common cell surface antigen markers of PSC are formed by elements of these structures or their terminal modifications. The antigens, SSEA-4 and SSEA-3, are formed from the globoseries structures. The monoclonal antibody, MC813-70, defining SSEA-4, requires a terminal sialic acid, whereas the monoclonal antibody, MC631, defining SSEA-3 binds whether or not sialic acid is present. The epitopes of the blood group antigens, P and p^k^, defined by traditional antisera have been identified with the shorter terasaccharide and trisaccharide, respectively. Mouse cells, but not human cells can also add a terminal N-acetylgalactosamine to form the Forssman antigen. The epitopes of SSEA-1 and SSEA-5 are formed by the addition of fucose to a type 2 or type 1 poly-N-acetyl-lactosamine, which may be present as a glycolipid or as a glycoprotein. The epitopes of TRA-1-60 and TRA-1-81, and other related antigens, appear to be formed by the terminal addition of a type 1 N-acetyl-lactosamine disaccharide to the type 2 poly N-acetyl-lactosamine of keratin sulfate. One of two reported forms of TRA-1-60 also requires the presence of sialic acid, though the exact linkage remains to be determined. Fuc, fucose; Gal, galactose; Glc, glucose; GlcNAc, N-acetylglucosamine; GalNAc, N-acetylgalactosamine; NeuNAc, N-acetylneuraminic acid (sialic acid).

Stage-specific embryonic antigen-1 (SSEA-1) was defined by Davor Solter and Barbara Knowles (Solter and Knowles, 1978) using a monoclonal antibody, MC480, produced after immunizing a mouse with F9 murine EC cells. Like the F9 antigen, SSEA-1 is a cell surface antigen expressed generally on all murine EC cells studied and on the inner cell mass at the blastocyst stage of mouse development. Nevertheless, SSEA-1 is expressed by a variety of other cell types, including primordial germ cells and hematopoietic cells (Fox et al., 1981, 1983). Subsequent structural studies showed that the epitope of SSEA-1 is a carbohydrate structure of the Lewis blood group series, specifically Lewis-X, which is formed from the disaccharide, fucose(α1→3)N-acetylglucosamine, associated with a type 2 polylactosamine chain, itself formed from repeating disaccharides, galactose(β1→4)N-acetylglucosamine (Gooi et al., 1981) (Figure 1). The epitope is associated with both glycolipids, notably ceramides (Kannagi et al., 1982), and high molecular weight glycoproteins (Andrews et al., 1982b; Childs et al., 1983). It seems likely that this carbohydrate structure is also one of the principal antigens detected by the original anti-F9 serum (Gachelin et al., 1982).

Some early reports of cell lines derived from human teratocarcinomas suggested that, like mouse EC cells, human EC cells also express the F9 antigen (Holden et al., 1977; Hogan et al., 1977). However, a detailed comparison of several human teratocarcinoma cell lines, and the xenograft tumors that they formed, concluded that human EC cells differ from mouse EC cells and do not express SSEA-1 (Andrews et al., 1980). Then, a second antigen, SSEA-3, was defined by the Solter and Knowles group (Shevinsky et al., 1982) with a monoclonal antibody, MC631, produced from a rat immunized with 4-cell cleavage stage mouse embryos. This antigen is expressed by cleavage stage mouse embryos, but not by the inner cell mass, nor by mouse EC cells. In contrast to SSEA-1, however, SSEA-3 is expressed by human EC cells in germ cell tumors (Damjanov et al., 1982) and in culture (Andrews et al., 1982a). A further antigen, SSEA-4, was then defined by a new monoclonal antibody, MC813-70, produced after immunizing a mouse with a human EC cell line (Kannagi et al., 1983). This antigen showed a very similar pattern of reactivity to SSEA-3, in both the mouse and human. Importantly, both SSEA-3 and SSEA-4 are downregulated upon differentiation of human EC cells, while SSEA-1 appears on some differentiated cells (Andrews et al., 1984b; Andrews, 1984; Fenderson et al., 1987). As predicted from these studies with human EC cells, when human ES cells were eventually isolated, these too proved to be different from mouse EC and ES cells, expressing SSEA-3 and SSEA-4, but not SSEA-1 (Thomson et al., 1998; Draper et al., 2002). Likewise, human iPS cells exhibit a similar cell surface antigen phenotype (Takahashi et al., 2007; Yu et al., 2007), so that SSEA-3 and SSEA-4 have both become widely accepted markers for all human PSCs (International Stem Cell Initiative, 2007).

Like SSEA-1, the epitopes of SSEA-3 and SSEA-4 are carbohydrates carried by glycosphingolipids. However, in this case, rather than lactoseries structures, the core carbohydrate chains are globoseries structures that are characteristically expressed in undifferentiated human EC cells (Kannagi et al., 1983; Wenk et al., 1994) (Figure 1). Of these, the SSEA-3 and SSEA-4 epitopes are formed by the sialyl-gal-globoside structure but, whereas SSEA-4 reactivity depends on the terminal sialic acid moiety, SSEA-3 reactivity does not. In fact, among the glycosphingolipids, the precursor for both the SSEA-1 lactoseries structures and the SSEA-3/-4 globoseries structures, as well as a series of gangliosides that appear on various differentiated derivatives, particularly in the nervous system, is the same, namely, lactosylceramide. The switch between a cell making lactoseries, globoseries, or ganglioseries core structures then appears to depend on how the precursor disaccharide is extended by rate-limiting enzymes that respectively add glucosamine, galactose, or sialic acid as the third sugar in the chain (Chen et al., 1989). In fact, the distinction between mouse and human EC or ES cells relates, in part, to the terminal modification of common core structures. Thus, mouse EC cells, like human EC cells, also produce globoseries structures but modify these by the addition of a terminal galactosamine moiety to form the Forssmann antigen (Willison et al., 1982). By contrast, humans lack the gene encoding this particular glycosyl transferase so that Forssmann antigen cannot be synthesized by human cells.

As with SSEA-1 in the mouse, the value of SSEA-3 and SSEA-4 as markers for human PSCs, and for monitoring their differentiation, is context dependent, since they are also widely expressed on a variety of other cell types, notably red blood cells where they correspond to the P blood group antigen, which is expressed by the red blood cells of almost all individuals (Tippett et al., 1986). There are, however, rare people who lack the glycosyl transferases required to add the third and fourth monosaccharides to the growing globoseries polysaccharide chain. These individuals do not express the P antigen on their red cells, which exhibit the so-called pp and p^k^ phenotypes (Race and Sanger, 1975) (Figure 1). Red cells from these people also lack SSEA-3 and SSEA-4 (Tippett et al., 1986). One might, therefore, expect PSCs from such people would also lack expression of SSEA-3 and -4, but to our knowledge no such lines have been reported. Rather more commonly than the pp and p^k^ phenotypes, some people (approximately 1% of Caucasian populations) lack another red cell antigen of the P blood group, called Luke (Race and Sanger, 1975). The Luke antigen seems to correspond with SSEA-4 since that, but not P antigen or SSEA-3, is absent from Luke negative red blood cells (Tippett et al., 1986). One might, therefore, expect PSCs from these individuals would also lack expression of SSEA-4, but not SSEA-3, but again, to our knowledge, no such lines have been reported.

Another carbohydrate surface antigen of human PSCs was recently identified and named SSEA-5 (Tang et al., 2011). Its epitope is formed by a disaccharide, fucose(α1→2)galactose, associated with a type 1 polylactosamine chain of repeating galactose(β1→3)N-acetylglucosamine disaccharides, in contrast with the type 2 polylactosamine core of SSEA-1 (Figure 1). As with the other SSEA markers, SSEA-5 is downregulated during human PSC differentiation, but its expression is not unique to human PSCs and it is also expressed by various somatic cells.

The function of the SSEA series of carbohydrate antigens remains obscure. Individuals with the pp and p^k^ phenotypes develop normally, although women with these phenotypes suffer high rates of early spontaneous abortion, which might be caused by an immune response to embryos expressing the SSEA-3, SSEA-4, and P antigens (Lopez et al., 1983; Tippett et al., 1986). Further, inhibition of the synthesis of glycosphingolipids in human EC cells or in medaka fish embryos by an inhibitor of glucosyl ceramide synthetase does not interfere with differentiation or embryonic development (Fenderson et al., 1992, 1993). Also, some clonal sublines of one pluripotent human EC cell line, TERA2, lack expression of SSEA-3 and -4, without affecting their ability to differentiate (Thompson et al., 1984; Andrews et al., 1985). However, intriguingly, when grown as xenograft teratocarcinomas in nude mice, and re-explanted back to *in vitro* culture, these cells reacquire permanent expression of these antigens, suggesting a link, albeit obscure, to their growth as tumors *in vivo* (Andrews et al., 1985). Nevertheless, despite the absence of any obvious function, and provided proper consideration is given to their expression on some differentiated cells, SSEA-3 and SSEA-4 remain invaluable markers for undifferentiated human PSCs. Importantly, though, their expression does not necessarily imply pluripotency since cells that have lost or have a diminished capacity to differentiate may still express these antigens (Andrews et al., 1982a, 1996; International Stem Cell Initiative, 2007).

### High-molecular-weight cell surface glycoproteins

Several studies have shown that both mouse and human EC cells express cell surface-associated, high-molecular-weight polysaccharide structure in contrast with many of their differentiated derivatives (Muramatsu et al., 1978; Muramatsu, 2017). For example, the F9 antigen and SSEA-1 may be associated with proteoglycans, as well as glycolipids (Andrews et al., 1982b; Childs et al., 1983; Gachelin et al., 1982). Several monoclonal antibodies that were produced against human EC cells have defined cell surface antigens that are associated with high-molecular-weight glycoproteins. These include TRA-1-60, TRA-1-81, and 8-7D (Andrews et al., 1984a), GCTM2 (Pera et al., 1988), and K4 and K21 (Rettig et al., 1985). They are all strongly expressed by undifferentiated human EC cells, including nullipotent lines, and show strong downregulation upon differentiation. Of these, TRA-1-60, TRA-1-81, and GCTM2 have also been shown to be widely expressed on human ES and iPS cells, and are widely used to monitor human PSC cultures (International Stem Cell Initiative, 2007). However, despite their developmental regulation during human PSC differentiation, these antigens are also expressed by a variety of diverse adult tissues, for example, parts of the gut and various blood vessels (Andrews et al., 1984a). Thus, they are not unique to undifferentiated PSCs.

The exact molecular structures of these antigens have still not been definitively characterized. The epitopes can all be present on the same high-molecular-weight glycoprotein(s) (Badcock et al., 1999), though TRA-1-60, TRA-1-81, and 8-7D, at least, can also be present on distinct molecules (Andrews et al., 1984a). With the possible exception of GCTM2, the epitopes are all oligosaccharide structures, and it is likely that they represent alternative or sequential glycosylation of a core protein, which has been suggested to be podocalaxyn and may be recognized by GCTM2 (Schopperle and DeWolf, 2007; Schopperle et al., 2003). Based on sensitivity to keratinase, we previously proposed that the TRA-1-60 epitope is a modification of keratan sulfate, which has a type 2 polylactosamine structure (Badcock et al., 1999). However, in recent detailed studies of oligosaccharides expressed by human PSCs, it was reported that the TRA-1-60 and TRA-1-81 epitopes include type1 lactosamine, though linked to type 2 polylactosamine chains (Natunen et al., 2011; Nakao et al., 2023). It is notable that the glycolipid antigen, SSEA-5, which is expressed by undifferentiated human PSCs, also has a type 1 polylactosamine structure, whereas SSEA-1, which is generally not expressed by human PSCs, is formed from a type 2 polylactosamine structure.

## Other cell surface proteins

Several other diverse proteins associated with the cell surface, and detectable as cell surface antigens, have also been used to mark undifferentiated PSCs. Among these, alkaline phosphatase (ALP) has a long history. This enzyme exists in several isoforms, a tissue non-specific isoform (TNALP) encoded in humans by a gene located on chromosome 1, and several tissue specific isoforms, including the intestinal, placental (PLAP), and placental-like, or germ cell (GCAP) isoforms, encoded in humans by a gene cluster on chromosome 2 (Millan, 2006; Sharma et al., 2014). First studied in mouse EC cells, ALP is readily detectable by staining based on its enzymatic activity and typically is downregulated upon differentiation (Bernstine et al., 1973). Similarly, human EC cells, whether pluripotent or nullipotent, express high levels of ALP (Andrews et al., 1980, 1996; Benham et al., 1981). In this case, at least two isoforms of ALP are expressed, the bulk of activity being associated with TNALP, while a smaller, variable proportion is associated with the placental-like, or GCAP isozyme that is expressed in primordial germ cells. Several monoclonal antibodies are now available to detect ALP as a cell surface antigen and two, TRA-2-49 and TRA-2-54 (Andrews et al., 1984c), have been used to confirm high-level expression of TNAP in undifferentiated ES cells (Draper et al., 2002; International Stem Cell Initiative, 2007) and in iPS cells (Takahashi et al., 2007). However, although downregulated upon differentiation, TNALP is also expressed by a wide variety of somatic cells, while the PLAP and GCAP isoforms are commonly re-expressed in tumors (Sharma et al., 2014).

CD90 and CD9 have been used from time to time as markers of undifferentiated human PSCs, in which both are strongly expressed (International Stem Cell Initiative, 2007; Andrews et al., 1983; Carpenter et al., 2004). They both show downregulation upon differentiation, but both antigens are also expressed by various somatic cells. Also, like the other markers, they are expressed by nullipotent EC cells, as well as pluripotent lines. CD9 is also strongly expressed in mouse ES cells and downregulated upon differentiation, though is not required for pluripotency (Oka et al., 2002; Akutsu et al., 2009). In contrast, Thy1 is not expressed at least by mouse EC cells (Stern et al., 1975), highlighting another difference between mouse and human PSC.

Testicular-derived growth factor (TDGF1) is another cell surface-associated protein that is also used as a marker for PSCs (International Stem Cell Initiative, 2007). Originally named CRIPTO, it is a member of the epidermal growth factor/transforming growth factor family of growth factors (Ciccodicola et al., 1989). There are a number of closely related sequences in the mouse and human genomes, some of which may be expressed (Liguori et al., 1996), so that it is important to validate the specificity of probes used for detecting its expression. TDGF1 is expressed by both mouse and human PSCs, including nullipotent EC cells, and in the blastocyst of mouse embryos. It is strongly downregulated upon differentiation, but it is also widely expressed in a variety of somatic tissues and tumors (Klauzinska et al., 2014). TDGF1 does not seem to be required for maintenance of undifferentiated PSC, but mouse ES cells in which TDGF1 expression has been knocked out are defective in cardiac differentiation, though able to form other mesoderm, endoderm, and ectodermal cell types (Xu et al., 1998).

## Transcription factors

Both mouse and human PSCs are characterized by the expression of several transcription factors that play a significant role in establishing and maintaining these cells. Indeed, vectors encoding several of these, notably OCT4 (POU5F1), SOX2, and NANOG, are widely used to promote reprogramming of somatic cells to a pluripotent state (Takahashi and Yamanaka, 2006; Takahashi et al., 2007; Yu et al., 2007). These three, in particular, have also become widely used as markers of undifferentiated PSCs. Nevertheless, despite their functional links to establishing and maintaining pluripotency, the expression of none of these necessarily indicates pluripotency.

OCT4, sometimes referred to as OCT3/4, and formally as POU5F1, is a POU domain transcription factor originally identified in oocytes, the early mouse embryo and primordial germ cells, and was first cloned from the mouse EC cell line, F9 (Scholer et al., 1989). Expressed generally in mouse PSCs, expression of OCT4 is required for the development of pluripotent cells in the blastocyst of mouse embryos (Nichols et al., 1998), while downregulation of OCT4 in mouse ES cells results in their differentiation (Niwa et al., 2000). Similarly, OCT4 is also widely expressed in human PSC (International Stem Cell Initiative, 2007) and its downregulation also results in their differentiation (Matin et al., 2004). Although there have been reports of expression of OCT4 in various somatic tumor cells, this might reflect expression of pseudogenes, of which there are many (Cantz et al., 2008). Nevertheless, in one report (Atlasi et al., 2008) one splice variant of OCT4, OCT4A, was found to be expressed in various tumor cells, whereas another isoform, OCT4B, was found to be restricted to human ES cells and EC cells, but irrespective of whether they retained pluripotency or were nullipotent.

NANOG was first identified in mouse ES cells as a putative factor that is required to establish and maintain pluripotency (Chambers et al., 2003; Mitsui et al., 2003). Unlike OCT4, it is not expressed in oocytes or cleavage stage embryos, but appears in the inner cell mass of the blastocyst (Hatano et al., 2005). Subsequently, it is downregulated in the somatic lineages (Hart et al., 2004). NANOG shows similar expression patterns in human PSC and embryos (Hart et al., 2004). Although less studied than OCT4, NANOG is expressed by nullipotent EC cells (International Stem Cell Initiative, 2007), and also in various stages of germ cell development (Hoei-Hansen et al., 2005; Yamaguchi et al., 2005) and so is not uniquely associated with pluripotency. If NANOG expression is eliminated in undifferentiated ES cells, the cells remain pluripotent, but have a much higher tendency to differentiation, suggesting that NANOG functions to stabilize the pluripotent state, though is not necessarily required for pluripotency itself (Silva et al., 2009).

SOX2 is one of a family of transcription factors identified from its relationship to the SRY gene, and containing the high mobility domain, SOX (SRY-Box) (Gubbay et al., 1990). It is expressed in the pluripotent cells of the mouse blastocyst and in mouse and human ES cells, where it plays a key role in regulating the pluripotent state (Avilion et al., 2003; Fong et al., 2008). Nevertheless, it is also widely expressed and functions in various somatic cells, notably in the nervous system (Uwanogho et al., 1995; Ellis et al., 2004), and cancer cells (e.g., Li et al., 2004).

## Discussion

Pluripotency is a term that is defined by function so that, ultimately, a PSC can only be identified by its ability to differentiate into all the somatic cells of an organism. Accordingly, as summarized in the recent report of the ISSCR on “Standards for Human Stem Cell Use in Research” (https://www.isscr.org/standards), a PSC line can only be characterized as pluripotent if this property is demonstrated functionally by appropriate differentiation assays. Yet, for routine monitoring of cell lines that have been already identified as pluripotent, or for assessing the progress of differentiation in particular experiments, or for identifying likely PSC during initial isolation, such as the preparation of large panels of iPSC, the markers discussed in this review have proved to be invaluable tools. These particular markers are by no means the only markers that can be used, but they do represent those that have found wide acceptance (International Stem Cell Initiative, 2007). Greater precision might be achieved using multiple markers or bioinformatic analysis of transcriptome data, but pluripotency can only be definitively confirmed by functional assays (International Stem Cell Initiative, 2018).

PSCs in culture are commonly heterogeneous with respect to patterns of gene expression and behavior. For example, we have identified transient PSC substates that exhibit biases in their patterns of differentiation (Allison et al., 2018; Stavish et al., 2020). Other states of pluripotency have been identified corresponding to different developmental stages in the early embryo from cells of the inner cell mass, which have been designated ‘naive’, through to a so-called primed state (Nichols and Smith, 2009). Cells in these different developmental states, while still exhibiting pluripotency defined by their capacity to differentiate, may show differences in their patterns of gene activity, growth behavior, or requirements for different growth factors. Generally, they express the markers that we have discussed in this review, though there may be subtle differences in their levels of expression. However, markers to characterize and define different states, notably naive and primed, have been proposed and have been discussed in detail in a recent review by Collier and Rugg-Gunn (2018).

The utility of the markers commonly used for monitoring PSCs is not dependent on our knowledge of their function in pluripotency. Indeed, in most cases their function is obscure. Rather, their value comes from the close correlation of a pluripotent phenotype with their expression. However, the opposite is not true: in no case does their expression necessarily correlate with pluripotency, since many are widely expressed by various other cells or by derivatives of PSCs that have lost the ability to differentiate, nullipotent stem cells. Only the core transcription factors OCT4 and SOX2 appear to have a definite role in establishing and maintaining pluripotency, since their expression is required for reprogramming somatic cells to a pluripotent state while their knock-out or downregulation typically results in differentiation. Nevertheless, even these are typically expressed by nullipotent stem cells as well as other cell types, such as primordial germ cells (OCT4) or neural stem cells (SOX2); their expression may be required for pluripotency, but it is not sufficient. Consequently, claims for the presence of pluripotent cells in adult tissues based solely on the expression of markers, without functional evidence, are likely incorrect.

The use of single molecules, whether complex carbohydrates, proteins, or RNA transcripts to monitor cell types has a long history and has contributed much to our understanding of PSC. However, none of those currently available are markers of pluripotency itself. Rather they are typically expressed, but not uniquely, by cells that can be shown to be pluripotent. A proper interpretation of the information they provide demands a consideration of their nature and their patterns of expression in cells beyond those that are demonstrably pluripotent.
